# Impact of Primary Percutaneous Coronary Intervention on Complete Atrioventricular Block With Acute Inferior ST-Elevation Myocardial Infarction

**DOI:** 10.7759/cureus.10013

**Published:** 2020-08-25

**Authors:** Jahanzeb Malik, Talha Laique, Muhammad Hasan Farooq, Umar Khan, Farhan Malik, Muhammad Zahid, Atif Majid

**Affiliations:** 1 Cardiology, Rawalpindi Institute of Cardiology, Rawalpindi, PAK; 2 Pharmacology, Lahore Medical and Dental College, Lahore, PAK; 3 Internal Medicine, Ysbyty Gwynedd Hospital, Bangor, GBR; 4 Pulmonary Medicine, University Hospital Limerick, Limerick, IRL; 5 Internal Medicine, Blackpool Teaching Hospitals National Health Service Foundation Trust, Blackpool, GBR; 6 Family Medicine, Sherbourne Medical Center, Sherbourne, GBR; 7 Paediatrics and Child Health, Aukland City Hospital, Aukland, NZL

**Keywords:** complete av block, inferior stemi, acute coronary syndrome

## Abstract

Background and aims

Complete atrioventricular block (CAVB) is associated with poor clinical outcomes in ST-elevation myocardial infarction (STEMI). This study determined the frequency and outcomes of primary percutaneous coronary intervention (PPCI) in patients with CAVB with acute inferior STEMI.

Methods

We conducted an observational, prospective study and enrolled 151 patients who were diagnosed with inferior STEMI. All patients received PPCI. The clinical outcomes were compared in patients with and without CAVB. The data was recorded on a collection form and analyzed on Statistical Package for Social Sciences (SPSS) software. Descriptive statistics were applied. For quantitative variables, standard deviation and mean were obtained, and statistical tests were also applied.

Results

Baseline characteristics were homogeneous in all patients. Half of the study population was either diabetic or hypertensive. Out of 151 participants, 21 (13.9%) developed CAVB. Two-thirds of the patients, who had developed heart block, reverted after PPCI. After a follow-up of two weeks, in-hospital mortality did not differ between the groups.

Conclusion

We conclude that PPCI can improve outcomes of CAVB-complicated acute inferior STEMI and suggest that primary PCI should be the preferred reperfusion therapy in patients with CAVB with STEMI.

## Introduction

Myocardial infarction (MI) can cause irreversible cell death of the heart muscle by prolonged ischemia. It occurs due to an imbalance in oxygen supply and demand owing to plaque rupture with thrombus formation in the coronary artery. The risk factors include hypertension, smoking, obesity, diabetes, and dyslipidemia [1]. The infarct depends on the artery which has been occluded. Inferior wall MI usually occurs after occlusion of the dominant right coronary artery (RCA) or left circumflex artery (LCX) [2].

Inferior infarcts are responsible for poor outcomes [3]. An inferior MI may diminish blood supply to the atrioventricular node (AV), causing a complete atrioventricular block (CAVB). In this case, it is usually temporary and typically resolves within two weeks [4]. CAVB can be preceded by hemiblocks [5].

There are some mechanisms that have been explained. AV node is supplied by the nodal branch, often arising from the right coronary artery. Severed blood supply to the node causes malfunction of the myocardial circuits, causing variable blocks. Another proposed mechanism is the high vagal tone due to the Bezold-Jarisch reflex [6].

In patients with acute coronary syndromes, primary percutaneous coronary intervention (PPCI) can be life-saving as it reduces major adverse cardiovascular events if compared with medical treatment [7]. With the advent of PPCI, the alarming incidence of CAVB is reduced [8]. CAVB, if persistent after PPCI, can be treated with a pacemaker.

The purpose of conducting the study is to determine the burden of CAVB in patients with inferior infarcts and its outcome in terms of resolution or persistence of CAVB and in-hospital mortality after primary PCI so that the patient can be managed accordingly. 

## Materials and methods

This observational, prospective study was conducted at our institute for a duration of six months from January 2020 to July 2020. The sample size was calculated using the Statistical Package for Social Sciences (SPSS) software. It was found to be 151. The sampling method was non-probability consecutive sampling.

Permission was sought from the hospital ethical committee. Written consent was taken from the patients. Particulars of all the patients who met the inclusion and exclusion criteria were recorded in the data collection form.

Patients presenting with chest pain had their electrocardiograms done. Upon meeting the criteria of inferior MI, consent for PPCI was taken and permission for enrollment in this study group was taken from the family. ST-elevation of more than 2 mm for males and 1.5 mm for females was taken as inferior MI in two or more contiguous leads (II, III, arteriovenous fistula (aVF)). Any conduction abnormality was recorded at this point in time. After primary PCI, patients were shifted to post-catheterization wards and monitored for any CAVB. Patients who did not have any conduction abnormalities were discharged after 24 hours. A temporary pacemaker was placed by a resident cardiologist during primary PCI in patients with CAVB, and they were observed for two weeks for the conduction abnormality to settle. In-hospital mortalities were included in the study.

The data collected was analyzed using IBM's Statistical Package for Social Sciences version 26. Mean and standard deviation was calculated for quantitative variables such as age and body mass index (BMI). Frequency and percentage were calculated for qualitative variables. Stratification was used to control effect modifiers such as age, gender, and co-morbid conditions. A chi-square test was also applied, and a p-value of less than 0.05 was considered significant.

## Results

This study included 151 patients with inferior myocardial infarction. The demographic details and co-morbid conditions are shown in Table 1.

**Table 1:** Demographic details, CAVB status, and Deaths Diabetes (DM); Hypertension (HTN); Chronic kidney disease (CKD); Complete Atrioventricular block (CAVB); Permanent Pacemaker (PPM)

Demographics and comorbid conditions	
Age	57±11.34
Gender (n,%)	Males: 129(85.4%)
	Females: 22(14.5%)
DM (n,%)	73(48.3%)
HTN (n,%)	63(41.7%)
Smoking (n,%)	64(41.1%)
CKD (n,%)	5(3.3%)
Dyslipidemia (n,%)	69(45.7%)
CAVB	21 (13.9%)
CAVB resolved	14(66.6%)
PPM inserted	5(71.4%)
Deaths	5(3.3%)

There were 21 (14%) patients who developed CAVB and 130 (86%) patients did not. In 14 patients (66.6%) CAVB reverted to normal sinus rhythm while five (71.4%) patients had a permanent pacemaker inserted. There were five (3.3%) in-hospital deaths, of which two had CAVB. None of the characteristics found was statistically significant with CAVB.

## Discussion

CAVB is a known complication of acute MI. In particular, patients with inferior MI develop CAVB in increased frequency as compared with other patients. STEMI increases the risk of CAVB compared with patients with non-STEMI [9].

In this article, we studied the frequency of CAVB and its outcome after PPCI. The in-hospital mortality rates and major adverse cardiac events did not differ significantly in patients with and without CAVB. Several studies have shown that patients with inferior MI who are complicated by CAVB had an elevated risk of in-hospital events [10,11]. In one study, CAVB significantly influenced the outcome when associated with right ventricular infarction, leading to high mortality owing to cardiomyopathy [12].

In our study, the mean age of the patients was 57±11.34 years. Most of the patients were males (85.4%). Seventy-three (48.3%) of the participants were diabetic and 41.7% of the participants were hypertensive. This was relatively similar to a study that described an older population that was predominantly male [13]. In one study, the co-morbid conditions associated with poor outcomes were diabetes and hypertension [14]. This was not the case in our study.

There is a likely mechanism that has been explained for both diabetes and hypertension. Both diseases damage the blood vessels and therefore predispose patients to cardiac autonomic neuropathy [15]. Vasoconstriction in damaged vessels causes changes in the secondary messenger system which ultimately leads to fibrosis of the conducting tree. The end result is a longer refractory period within the ventricles which causes inhomogeneity of the refractory period and repolarization, leading to heart block [16].

In our setting, 21 (13.9%) patients developed CAVB. After a primary percutaneous intervention, 14 (66.6%) patients reverted to sinus rhythm, while seven (33.34%) did not. This frequency of CAVB was slightly higher when compared to another study in which the frequency was 11.3% [17]. In our study, the in-hospital mortality was only 3.3%, which is significantly lower than in a previous study [11].

There were several limitations to our study. This was a single-centre study with a small sample size. Extensive patient characteristics were not included and the type of pacemakers was not analyzed. Follow-up time was short and only in-hospital mortality was taken into account. The disease burden was not assessed in the patients.

## Conclusions

The incidence of the complete atrioventricular block (CAVB) complicating inferior ST-elevation myocardial infarction (STEMI) has reduced in the primary percutaneous coronary intervention (PPCI) era. In conclusion, primary PCI can ameliorate CAVB in acute inferior STEMI with an acceptable rate of in-hospital mortality. It should therefore be the primary reperfusion therapy in such patients.
